# Association between statin use on delirium and 30-day mortality in patients with chronic obstructive pulmonary disease in the intensive care unit

**DOI:** 10.1186/s40001-023-01551-3

**Published:** 2023-12-08

**Authors:** Jiangling Xia, Chunhuan Hu, Leilei Wang, Yuzhu Zhang

**Affiliations:** 1 Department of Anesthesiology, Zibo Central Hospital, No. 54 Gongqingtuan Road, Zibo, Shandong China; 2 Department of Anesthesiology, The Second Affiliated Hospital of Mudanjiang Medical College, Mudanjiang, Heilongjiang China; 3 School of Architecture and Engineering, Zibo Vocational Institute, Zibo, Shandong China

**Keywords:** Delirium, Mortality, Chronic obstructive pulmonary disease, Statin, Propensity analysis

## Abstract

**Background:**

Delirium occurs frequently in patients with chronic obstructive pulmonary disease in the intensive care unit. Effective prevention and treatment strategies for delirium remain limited. We aimed to assess delirium and 30-day mortality in patients with chronic obstructive pulmonary disease who were statin and non-statin users.

**Methods:**

In this retrospective study, patients with chronic obstructive pulmonary disease were identified from the Medical Information Mart for Intensive Care database (MIMIC-IV). The primary exposure variable was the use of statins 3 days after entering the intensive care unit and the primary outcome measure was the presence of delirium. The secondary outcome measure was 30-day mortality. Since the cohort study was retrospective, we used an inverse probability weighting derived from the propensity score matching to balance different variables.

**Results:**

Among a cohort of 2725 patients, 1484 (54.5%) were statin users. Before propensity score matching, the prevalence of delirium was 16% and the 30-day mortality was 18% in patients with chronic obstructive pulmonary disease. Statin use was significantly negatively correlated with delirium, with an odds ratio of 0.69 (95% CI 0.56–0.85, *p* < 0.001) in the inverse probability weighted cohort and 30-day mortality of 0.7 (95% CI 0.57–0.85, *p* < 0.001).

**Conclusions:**

Statin use is associated with a lower incidence of delirium and 30-day mortality in patients with chronic obstructive pulmonary disease in the intensive care unit.

**Supplementary Information:**

The online version contains supplementary material available at 10.1186/s40001-023-01551-3.

## Introduction

Chronic obstructive pulmonary disease (COPD) is a common, preventable, and treatable disease, which is characterized by persistent respiratory symptoms and airflow limitations due to airway and/or alveolar abnormalities usually caused by significant exposure to dangerous particles or gases. Exacerbations negatively impact health status, hospitalization rates, and disease progression [1]. COPD is currently the third leading cause of deaths worldwide [2]. Many patients in the intensive care unit (ICU) present with comorbid COPD. Delirium is common in patients with COPD and in those with respiratory failure receiving mechanical ventilation [3]. Furthermore, the probability of survival in patients with COPD undergoing coronary artery bypass grafting (CABG) who developed postoperative delirium is significantly lower [4]. Delirium manifests itself as acute state of attention, cognitive impairment and mental disorder that can be related to potential physiological disorders [5]. Delirium is often accompanied by increased morbidity, prolonged hospitalization, higher medical costs and mortality [6]. However, the treatment of delirium is limited, so prevention is critical.

Recently, contradictory evidence has been provided on the role of statins in preventing delirium. Some studies have found that statins can reduce the occurrence of delirium, including in the ICU and in cases of postoperative delirium [7, 8]. Other studies have contrary findings that the effect of statins on delirium is related to the severity of the disease and not to its occurrence [9, 10]. A meta-analysis that included both cohort studies and randomized clinical trials (RCT) showed that treatment with statins was associated with a significant reduction in all-cause mortality in patients with COPD [11]. Although a meta-analysis that included only RCTs did not show benefits for patients with COPD [12, 13]. The role of statins on mortality in patients with COPD is controversial.

Systemic and respiratory inflammation is believed to be the major cause of lung damage in COPD [14] and studies have shown that statins reduce serum levels of pro-inflammatory cytokines in patients with COPD [11] which were strong predictors of delirium [15]. As statins have a potent anti-inflammatory effect, the hypothesis that pharmacological intervention with statins can decrease the risk of delirium in patients with COPD needs to be confirmed. To date, no studies have examined the effects of statin use on delirium in patients with COPD. We used the Medical Information Mart for Intensive Care IV (MIMIC-IV) database (version 2.2) to investigate the relationship between statins and delirium and 30-day mortality in patients with COPD admitted to the ICU. In this study, we use weighted analysis according to the propensity score. Propensity score matching (PSM) was used to appropriately adjust for confounding factors, reduce the impact of these deviations and confounding variables, to allow a more reasonable comparison between the statin-exposed group and non-statin-exposed group.

## Methods

### Data sources

This retrospective cohort study was based on the MIMIC-IV database (version 2.2) [16], which contains data from patients admitted to the ICU of the Beth Israel Deaconess Medical Centre in Boston, Massachusetts, between 2008 and 2019. One author (JLX) obtained access to the database and was responsible for data extraction. The establishment of the MIMIC-IV database was approved by the institutional review boards of Beth Israel Deaconess Medical Centre and the Massachusetts Institute of Technology Affiliates. The informed consent requirement was waived because the data from all patients in the database were anonymized.

### Study population and data extraction

We included all patients who were first admitted to the ICU diagnosed with COPD [17] in the MIMIC-IV database. We excluded (i) patients with dementia; (ii) patients younger than 18 years; (iii) patients without a CAM-ICU estimate; (iiii) patients with a duration of stay in the ICU stay < 2 days; (v) patients with mild cognitive impairment (MCI). Navicat Premium (version 16.0) was used to extract raw data relative to patients diagnosed with COPD from the MIMIC-IV database (version 2.2). The data extracted included demographics, laboratory tests results, vital signs, comorbidities, and administered drugs. The following demographic information was extracted: age, sex, length of hospitalization, and duration of stay in the ICU. Vital signs such as systolic blood pressure (SBP), diastolic blood pressure (DBP), heart rate (HR), respiratory rate (RR), and oxygenated hemoglobin saturation (SpO_2_) were collected. Comorbidities including diabetes, asthma, coronary artery disease (CAD), peripheral vascular disease (PVD), congestive heart failure (CHF), malignant cancer, cerebrovascular disease (CVD), liver disease, and renal disease were extracted. Laboratory data included white blood cell (WBC) count, hemoglobin (HGB) level, platelets (PLT), blood glucose, anion gap, potassium, sodium, and calcium levels. We also extracted whether the patient received mechanical ventilation. The use of vasoactive drugs (norepinephrine, vasopressin, epinephrine) and antibiotics was also included. Whether the patient received oral or intravenous glucocorticoids was also determined. Whether the patient received ACEI/ARB, β-blockers, and statins 3 days after entering the ICU were extracted. A Simplified Acute Physiology Score (SAPS II), Charlson Comorbidity Index (CCI), and the Oxford Acute Severity of Illness Score (OASIS), which represents the severity of the disease, were also included.

### Statin use

We defined patients with records of 3 days of statin use before or after admission to the ICU as statin-exposed and others as non-statin-exposed. We searched drug ILIKE “statin” and NOT ILIKE “nystatin”, “mycostatin”, “imipenem-cilastatin”, “pentostatin”, and “sandostatin” in Navicat Premium (version 16.0). The medication prescriptions were recorded in the MIMIC-IV table (version 2.2) under “mimic-hospital, prescription”.

### Outcomes

The primary outcome was the occurrence of delirium during the stay in the ICU. The secondary outcome was the 30-day mortality rate. The Confusion Assessment Method for the Intensive Care Unit (CAM-ICU) method was used to assess delirium in patients [18].

### Statistical analysis

The characteristics of the patients are described in general and by group (statin-exposed and non-statin-exposed). The measured data are expressed as mean (standard deviation) or median (interquartile interval) according to whether they were normally distributed. A one-way analysis of variance (ANOVA) or Kruskal–Wallis H test was performed depending on whether the data were normally distributed. Categorical variables were expressed as percentages and treated with Chi-square tests.

Propensity score matching (PSM) was used to adjust for confounders between the non-statin and statin groups. The following prognostic variables related to the outcome at* p* < 0.2 in the univariate analysis (Additional file 1: Table S1) were included in the propensity score: age, HGB, WBC, sodium and calcium, CAD, asthma, liver disease, malignant cancer, SBP, DBP, HR, and SpO_2_ on the first day of admission, ACEI/ARB, antibiotic and glucocorticoids after admission to the ICU, and norepinephrine, vasopressin, epinephrine, SAPII, OASIS score and length of ICU stay for delirium were forced in the PSM. The variables that were included in the 30-day mortality analysis are shown in Additional file 1: Table S2. Using the estimated propensity scores as weights, an inverse probability weighting (IPW) model was used to generate a weighted cohort [19]. The probability of being exposed to statin was estimated using a logistic regression for each patient. We matched the two groups in a 1:1 ratio with a caliper width of 0.2. The standardized mean difference (SMD) was used to examine the degree of PSM. The R software packages (http://www.R-project.org, The R Foundation) and Free Statistics software versions 1.7 were used to perform all statistical analyses. Statistical differences were considered significant at *p* < 0.05.

### Sensitivity analysis

Patients were re-grouped according to whether they were on statins or not before ICU admission. There were three groups, no statin users, statin use after ICU admission and statin use both before and after ICU admission. There were no patients who were statin users before ICU and not statin users after ICU admission. Multivariate logistic regression analysis was used to ascertain the relationship between statin use and the incidence of delirium in patients with COPD adjusting covariates as for PSM. We did a subgroup analysis in logistic regression to investigate the association between statin use and delirium and 30-day mortality, as it differed between various subgroups classified by age, sex, CAD, CHF, antibiotic and glucocorticoids use, CCI, SAPII, OASIS score and length of ICU stay.

## Results

### Baseline characteristics

**Fig. 1:**
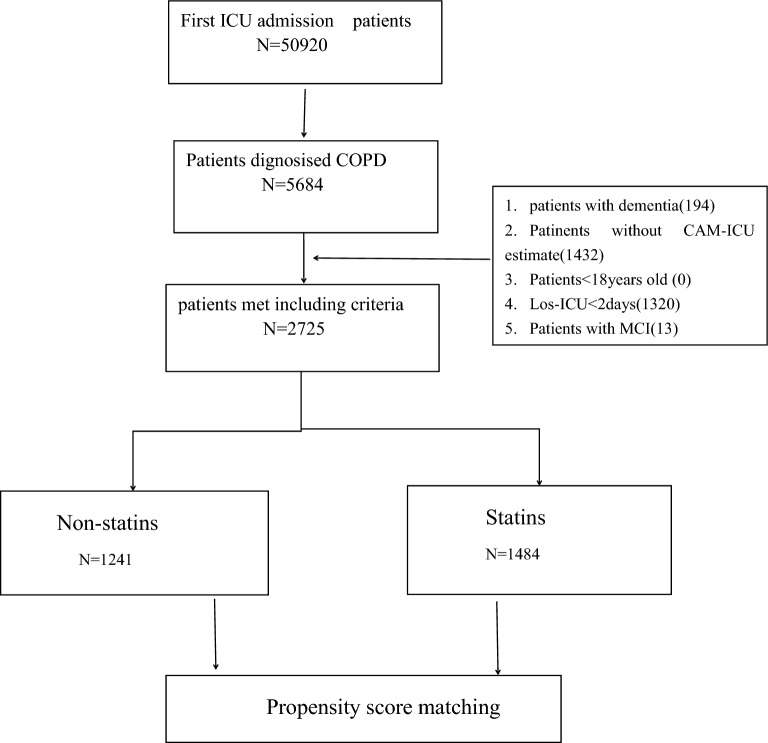
Flowchart of cohort selection

Among the 50,920 patients who were admitted to the ICU and included in the MIMIC-IV database, 2725 patients had COPD and were evaluated using CAM-ICU methods. The patient selection process is shown in Fig. 1. Table 1 summarizes the characteristics of the statin and non-statin groups. In total, 1484 (54.5%) patients were exposed to statins. The median age of the patients was 72 years (range, 64 to 80), and 53.4% were male. The total incidence of delirium was 16% (436/2725) and that of 30-day mortality was 18% (492/2725). Patients in the statin group had a higher age, a higher rate of diabetes, CAD, CHF, PVD, renal disease, and CVD. Moreover, they also received treatment with more ACEI/ARB and vasoactive drugs than those in the non-statin group (all* p* < 0.05). However, they had a lower use of antibiotics and glucocorticoids and the incidence of delirium (13.4–19.1%) and 30-day mortality was lower (15–21.7%) (for all* p* < 0.05).

**Table 1:** Baseline characteristics

Variables	Total (*n* = 2725)	Non-statins	Statins	*p*
		(*n* = 1241)	(*n* = 1484)	
Number				
Age(year)	72 (64, 80)	69 (61, 78)	73 (67, 80)	< 0.001
Gender(male)	1456 (53.4)	643 (51.8)	813 (54.8)	0.121
Los-ICU	5.5 ± 5.9	6.0 ± 6.4	5.1 ± 5.4	< 0.001
Hospital days	12.4 ± 10.2	12.9 ± 10.9	12.0 ± 9.7	0.013
Vitals				
SBP (mmHg)	117.0 ± 16.0	116.6 ± 15.6	117.4 ± 16.3	0.197
DBP (mmHg)	61.9 ± 10.8	63.1 ± 11.0	60.9 ± 10.6	< 0.001
HR (beats/min)	85.9 ± 15.6	87.7 ± 16.4	84.4 ± 14.7	< 0.001
RR (beats/min)	19.8 ± 3.7	19.9 ± 3.9	19.8 ± 3.5	0.29
SpO_2_ (%)	96.2 ± 2.3	96.2 ± 2.3	96.2 ± 2.2	0.826
Comorbidities, *n* (%)				
Asthma	114 ( 4.2)	55 (4.4)	59 (4)	0.544
CAD	456 (16.7)	117 (9.4)	339 (22.8)	< 0.001
CHF	1216 (44.6)	422 (34)	794 (53.5)	< 0.001
PVD	517 (19.0)	168 (13.5)	349 (23.5)	< 0.001
CVD	433 (15.9)	161 (13)	272 (18.3)	< 0.001
Diabetes	938 (34.4)	315 (25.4)	623 (42)	< 0.001
Liver disease	314 (11.5)	219 (17.6)	95 (6.4)	< 0.001
Renal disease	692 (25.4)	215 (17.3)	477 (32.1)	< 0.001
Malignant cancer	446 (16.4)	255 (20.5)	191 (12.9)	< 0.001
Laboratory events				
WBC(10^9^/L)	14.7 ± 11.2	15.0 ± 14.1	14.5 ± 8.0	0.249
HGB (g/L)	11.3 ± 2.2	11.5 ± 2.3	11.3 ± 2.1	0.014
PLT (10^9^/L)	147.0 (119.0, 196.0)	144.0 (118.0, 190.0)	149.0 (120.0, 199.2)	0.038
Glucose (mg/dL)	209.0 (156.0, 277.0)	206.0 (148.0, 284.0)	210.5 (160.8, 272.0)	0.184
Calcium (mg/dL)	8.6 ± 0.7	8.6 ± 0.8	8.6 ± 0.7	0.047
Sodium (mmol/L)	139.6 ± 4.9	139.5 ± 5.3	139.7 ± 4.6	0.342
Potassium (mmol/L)	4.7 ± 0.9	4.7 ± 0.9	4.7 ± 0.8	0.553
Anion gap (mmol/L)	16.4 ± 4.5	16.5 ± 4.7	16.3 ± 4.4	0.161
Treatment, *n* (%)				
Glucocorticoid	968 (35.5)	483 (38.9)	485 (32.7)	< 0.001
Antibiotic	2280 (83.7)	1073 (86.5)	1207 (81.3)	< 0.001
Vasopressin	284 (10.4)	114 (9.2)	170 (11.5)	0.054
Norepinephrine	575 (21.1)	238 (19.2)	337 (22.7)	0.024
Epinephrine	130 ( 4.8)	56 (4.5)	74 (5)	0.563
ACEI/ARB	416 (15.3)	122 (9.8)	294 (19.8)	< 0.001
β-blocker	1437 (52.7)	545 (43.9)	892 (60.1)	< 0.001
VENT	873 (32.0)	387 (31.2)	486 (32.7)	0.383
Scores				
CCI	7.4 ± 2.6	7.0 ± 2.6	7.8 ± 2.5	< 0.001
SAPII	39.8 ± 12.8	40.4 ± 14.0	39.3 ± 11.6	0.023
OASIS	34.3 ± 9.0	35.5 ± 9.4	33.3 ± 8.5	< 0.001
Outcomes, *n* (%)				
Delirium	436 (16.0)	237 (19.1)	199 (13.4)	< 0.001
30-day mortality	492 (18.1)	269 (21.7)	223 (15)	< 0.001

Data are presented as mean (SD), medians [interquartile ranges] or numbers (percentages)

*SBP* systolic blood pressure, *DBP* diastolic blood pressure, *HR* heart rate, *RR* respiratory rate, *CAD* coronary artery disease, *CHF* congestive heart failure, *PVD* peripheral vascular disease, *CVD* cerebrovascular disease, *HGB* hemoglobin, *WB* white blood cell count, *PLT* platelets, *ACEI/ARB* angiotensin-converting enzyme inhibitors/angiotensin II inhibitors, *VENT* mechanical ventilation, *CCI* Charlson comorbidity index, *SAPS II* Simplified Acute Physiology Score, *OASIS* Oxford Acute Severity of Illness Score, *Los* length of stay

### Propensity score matching analysis

**Fig. 2:**
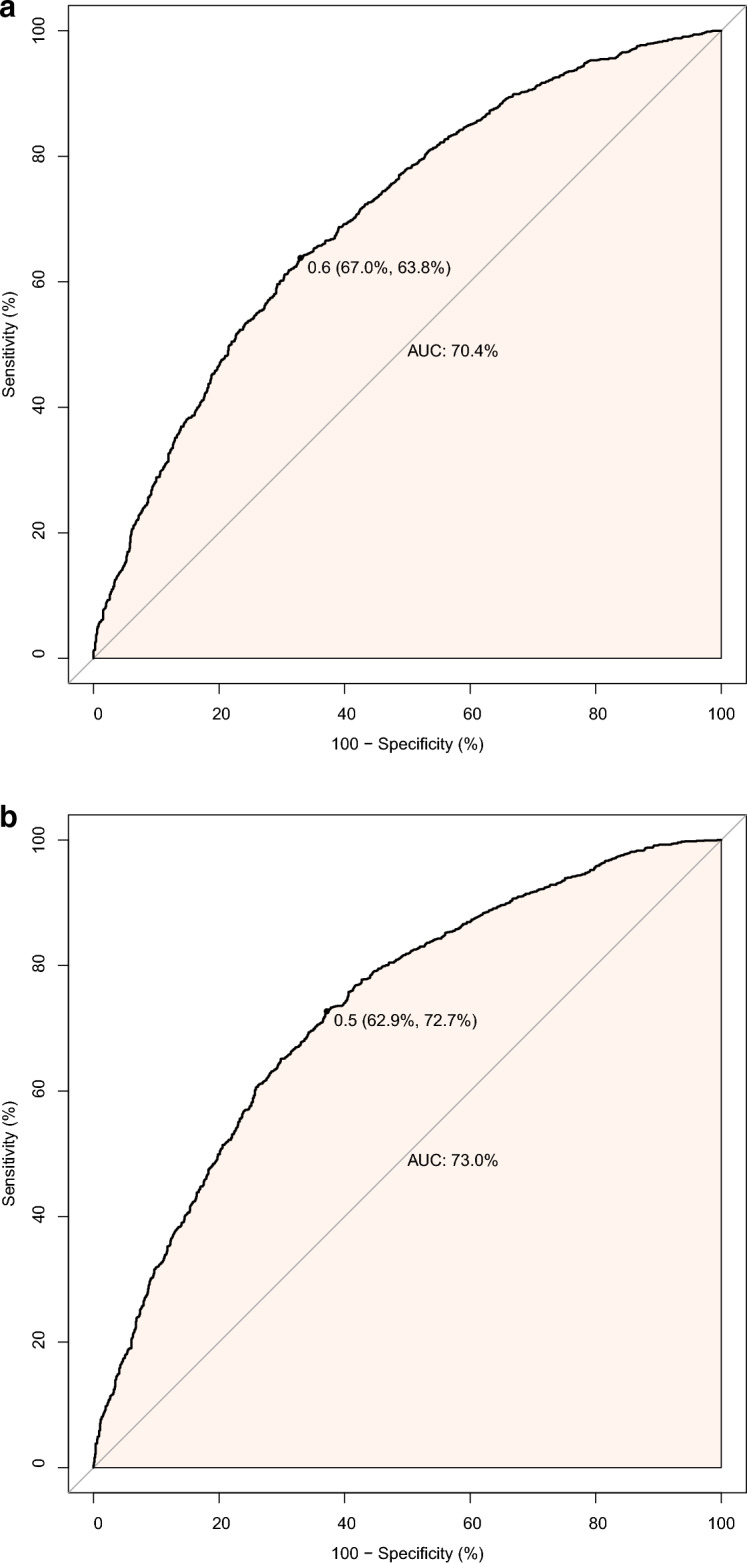
**A** Receiver operating characteristic (ROC) curve for delirium. **B** Receiver operating characteristic curve (ROC) for 30-day mortality

We used PSM to balance the baseline characteristics. After matching, 959 and 888 patients with delirium and 30-day mortality, respectively, were included in each group. The SMD of all covariates after matching was less than 0.1, indicating that the balance was sufficient after matching (Tables 2, 3). We also reported variables in the subject operating characteristic (ROC) curve for delirium (Fig. 2A) and 30-day mortality (Fig. 2B). The area under the curve (AUC) was calculated to assess the relationship between statin use and delirium (70.4%) and 30-day mortality (73%).

**Table 2:** Imbalance of patient characteristics before and after propensity score matching in the assessment of delirium

Item *n*	Unmatched			SMD	Matched			SMD
	Non-statins	Statins	SMD	− 0.1	Non-statins	Statins	SMD	− 0.1
	1241	1484			955	955		
Age (year)	69.46 (11.77)	73.12 (9.80)	0.338	> 0.1	71.56 (11.38)	71.54 (9.58)	0.002	< 0.1
SBP (mmHg)	116.61 (15.63)	117.41 (16.35)	0.05	< 0.1	117.03 (15.40)	117.40 (16.22)	0.023	< 0.1
DBP (mmHg)	63.14 (10.99)	60.91 (10.59)	0.207	> 0.1	62.22 (10.69)	62.15 (10.78)	0.006	< 0.1
HR (beats/min)	87.74 (16.36)	84.37 (14.75)	0.216	> 0.1	86.22 (15.95)	85.90 (15.38)	0.021	< 0.1
SpO_2_ (%)	96.16 (2.34)	96.18 (2.20)	0.008	< 0.1	96.15 (2.35)	96.18 (2.15)	0.009	< 0.1
Asthma	55 (4.4)	59 (4.0)	0.023	< 0.1	45 ( 4.7)	47 (4.9)	0.01	< 0.1
CAD	117 (9.4)	339 (22.8)	0.371	> 0.1	111 (11.6)	119 (12.5)	0.026	< 0.1
Liver disease	219 (17.6)	95 ( 6.4)	0.351	> 0.1	85 (8.9)	87 (9.1)	0.007	< 0.1
Malignant cancer	255 (20.5)	191 (12.9)	0.207	> 0.1	165 (17.3)	155 (16.2)	0.028	< 0.1
PLT (10^9^/L)	225.28 (114.45)	227.34 (99.49)	0.019	< 0.1	229.06 (108.70)	230.91 (105.12)	0.017	< 0.1
HGB (g/L)	11.46 (2.26)	11.26 (2.10)	0.094	< 0.1	11.38 (2.22)	11.42 (2.13)	0.018	< 0.1
Calcium (mg/dL)	8.58 (0.81)	8.64 (0.69)	0.076	< 0.1	8.62 (0.80)	8.61 (0.69)	0.012	< 0.1
Sodium (mmol/L)	139.55 (5.27)	139.73 (4.64)	0.036	< 0.1	139.70 (4.87)	139.72 (4.60)	0.005	< 0.1
Glucose (mg/dL)	169.56 (96.72)	177.37 (104.89)	0.077	< 0.1	170.52 (100.27)	172.39 (92.73)	0.019	< 0.1
Glucocorticoid	483 (38.9)	485 (32.7)	0.13	> 0.1	355 (37.2)	355 (37.2)	< 0.001	< 0.1
Antibiotic	1073 (86.5)	1207 (81.3)	0.14	> 0.1	802 (84.0)	804 (84.2)	0.006	< 0.1
Norepinephrine	238 (19.2)	337 (22.7)	0.087	< 0.1	202 (21.2)	188 (19.7)	0.036	< 0.1
Epinephrine	56 (4.5)	74 (5.0)	0.022	< 0.1	47 (4.9)	40 (4.2)	0.035	< 0.1
Vasopressin	114 (9.2)	170 (11.5)	0.075	< 0.1	102 (10.7)	88 (9.2)	0.049	< 0.1
ACEI/ARB	122 (9.8)	294 (19.8)	0.284	> 0.1	117 (12.3)	124 (13.0)	0.022	< 0.1
SAPII	40.39 (14.03)	39.28 (11.58)	0.087	< 0.1	39.81 (13.40)	39.74 (12.17)	0.006	< 0.1
OASIS	35.51 (9.39)	33.29 (8.46)	0.249	> 0.1	34.47 (9.08)	34.47 (8.60)	< 0.001	< 0.1
Los-ICU	6.01 (6.45)	5.10 (5.45)	0.152	> 0.1	5.51 (5.99)	5.55 (6.16)	0.006	< 0.1

An absolute MSD < 10% was considered to support the assumption of a balance between the groups. SMD: standardized mean differences. Data are presented as medians [interquartile ranges], mean [SD] or as numbers (percentages)

*SBP* systolic blood pressure, *DBP* diastolic blood pressure, *HR* heart rate, *CAD* coronary artery disease, *CVD* cerebrovascular disease, *RR* respiratory rate, *HGB* hemoglobin, *WB* white blood cell count, *ACEI/ARB* angiotensin-converting enzyme inhibitors/angiotensin II inhibitors, *SAPS II* Simplified Acute Physiology Score, *OASIS* Oxford Acute Severity of Illness Score, *Los* length of stay

**Table 3:** Imbalance of patient characteristics before and after propensity score matching in the assessment of 30-day mortality

Item *n*	Unmatched			SMD	Matched			SMD
	Non-statins	Statins	SMD	− 0.1	Non-statins	Statins	SMD	− 0.1
	1241	1484			882	882		
Age (year)	69.46 (11.77)	73.12 (9.80)	0.338	> 0.1	71.83 (11.33)	71.76 (9.87)	0.006	< 0.1
SBP (mmHg)	116.61 (15.63)	117.41 (16.35)	0.05	< 0.1	117.40 (15.67)	117.50 (16.32)	0.006	< 0.1
DBP(mmHg)	63.14 (10.99)	60.91 (10.59)	0.207	> 0.1	62.43 (10.97)	62.24 (10.85)	0.017	< 0.1
HR (beats/min)	87.74 (16.36)	84.37 (14.75)	0.216	> 0.1	86.25 (16.04)	85.81 (15.29)	0.028	< 0.1
RR (beats/min)	19.91 (3.87)	19.76 (3.48)	0.041	< 0.1	19.75 (3.67)	19.90 (3.54)	0.042	< 0.1
SpO_2_ (%)	96.16 (2.34)	96.18 (2.20)	0.008	< 0.1	96.18 (2.20)	96.15 (2.18)	0.013	< 0.1
CHF	422 (34.0)	794 (53.5)	0.401	> 0.1	374 (42.4)	377 (42.7)	0.007	< 0.1
Asthma	55 ( 4.4)	59 ( 4.0)	0.023	< 0.1	36 ( 4.1)	46 (5.2)	0.054	< 0.1
Diabetes	315 (25.4)	623 (42.0)	0.357	> 0.1	273 (31.0)	281 (31.9)	0.02	< 0.1
Liver disease	219 (17.6)	95 ( 6.4)	0.351	> 0.1	74 ( 8.4)	86 (9.8)	0.047	< 0.1
Renal disease	215 (17.3)	477 (32.1)	0.349	> 0.1	193 (21.9)	190 (21.5)	0.008	< 0.1
Malignant cancer	255 (20.5)	191 (12.9)	0.207	> 0.1	158 (17.9)	143 (16.2)	0.045	< 0.1
HGB(g/L)	11.46 (2.26)	11.26 (2.10)	0.094	< 0.1	11.37 (2.21)	11.47 (2.14)	0.046	< 0.1
WBC(10^9/L)	15.01 (14.11)	14.51 (7.95)	0.043	< 0.1	14.74 (10.07)	15.03 (8.22)	0.032	< 0.1
Glucose (mg/dl)	169.56 (96.72)	177.37 (104.89)	0.077	< 0.1	170.17 (100.76)	172.59 (99.14)	0.024	< 0.1
Sodium (mmol/L)	139.55 (5.27)	139.73 (4.64)	0.036	< 0.1	139.64 (5.04)	139.64 (4.51)	< 0.001	< 0.1
Potassium (mmol/L)	4.68 (0.90)	4.70 (0.83)	0.023	< 0.1	4.67 (0.89)	4.69 (0.80)	0.017	< 0.1
Anion gap (mmol/L)	16.50 (4.65)	16.26 (4.35)	0.054	< 0.1	16.16 (4.29)	16.22 (4.35)	0.014	< 0.1
Glucocorticoid	483 (38.9)	485 (32.7)	0.13	> 0.1	330 (37.4)	321 (36.4)	0.021	< 0.1
Antibiotic	1073 (86.5)	1207 (81.3)	0.14	> 0.1	741 (84.0)	742 (84.1)	0.003	< 0.1
Vasopressin	114 (9.2)	170 (11.5)	0.075	< 0.1	89 (10.1)	82 (9.3)	0.027	< 0.1
Epinephrine	56 (4.5)	74 (5.0)	0.022	< 0.1	44 (5.0)	43 (4.9)	0.005	< 0.1
Norepinephrine	238 (19.2)	337 (22.7)	0.087	< 0.1	181 (20.5)	171 (19.4)	0.028	< 0.1
ACEI.ARB	122 (9.8)	294 (19.8)	0.284	> 0.1	109 (12.4)	119 (13.5)	0.034	< 0.1
β-blocker	545 (43.9)	892 (60.1)	0.328	> 0.1	462 (52.4)	464 (52.6)	0.005	< 0.1
CCI	6.98 (2.62)	7.82 (2.47)	0.33	> 0.1	7.28 (2.65)	7.28 (2.47)	< 0.001	< 0.1
SAPII	40.39 (14.03)	39.28 (11.58)	0.087	< 0.1	39.57 (12.52)	39.78 (12.46)	0.017	< 0.1
OASIS	35.51 (9.39)	33.29 (8.46)	0.249	> 0.1	34.46 (8.84)	34.48 (8.79)	0.002	< 0.1
Los-ICU	6.01 (6.45)	5.10 (5.45)	0.152	> 0.1	5.48 (5.85)	5.42 (5.69)	0.01	< 0.1

An absolute MSD < 10% was considered to support the assumption of a balance between the groups. SMD: standardized mean differences. Data are presented as medians [interquartile ranges], mean [SD] or as numbers (percentages)

*SBP* systolic blood pressure, *DBP* diastolic blood pressure, *HR* heart rate, *RR* respiratory rate, *HGB* hemoglobin, *WB* white blood cell count, *ACEI/ARB* angiotensin-converting enzyme inhibitors/angiotensin II inhibitors, *CCI* Charlson comorbidity index, *SAPS II* Simplified Acute Physiology Score, *OASIS* Oxford Acute Severity of Illness Score, *Los* length of stay

### Association between statin exposure and outcomes

Before matching, regression analysis showed that statin exposure was significantly associated with a reduced odds ratio 0.66 (95% CI 0.53–0.81,* p* < 0.001) (Table 4) for delirium. After IPW, the risk of reduced delirium remained significantly associated with statin exposure with an odds ratio 0.69 (95% CI 0.56–0.85,* p* < 0.001). Before matching, the reduced risk of 30-day mortality was significantly related to statin exposure at an odds ratio of 0.64 (95% CI 0.53–0.78,* p* < 0.001). After IPW, the reduced risk of 30-day mortality remained significantly associated with statin exposure with an odds ratio of 0.7 (95% CI 0.57–0.85,* p* < 0.001). The results were similar to those obtained with the PSM model.

**Table 4:** Associations between statin use and the outcome in the crude analysis, multivariable analysis, and propensity-score analyses

Analysis	Delirium (%)	*p*	30-day mortality (%)	*p*
No. of events/no. of patients at risk (%)				
No statin use	237/1241 (19.1)		269 (21.7)	
Statin use	199/1484 (13.4)		223 (15)	
Crude analysis-hazard ratio (95% CI)	0.66 (0.53–0.81)	< 0.001	0.64 (0.53–0.78)	< 0.001
Multivariable analysis-hazard ratio (95% CI)	0.67 (0.52–0.85)	0.001	0.67 (0.52–0.85)	0.001
With matching	0.7 (0.54–0.9)	0.006	0.76 (0.6–0.97)	0.028
With inverse probability weighting	0.69 (0.56–0.85)	< 0.001	0.7 (0.57–0.85)	< 0.001
Adjusted for propensity score	0.7 (0.56–0.88)	0.002	0.73 (0.59–0.9)	0.003

*CI* confidence interval

Of all statin users, some used statins after ICU admission and some used statins both before and after ICU admission. We further subgrouped the patients using statins. Among the 1484 patients using statins, 644 patients started statins after ICU admission and 840 patients used statins both before and after ICU admission (Additional file 1: Table S5). Multifactorial regression showed that statin use after ICU admission can significantly reduced delirium regardless of statin use before ICU admission or not(*p* < 0.001 or *p* = 0.047). Statin use after ICU admission reduced 30-day mortality from 21.7% to 16.6% compared to non-statin use group, but it was not statistically significant (*p* = 0.287), which may require a large sample of studies for validation. Using statin before ICU admission can significantly reduce 30-day mortality (*p* < 0.001) (Table 5).

**Table 5:** Odds ratio for delirium and 30-day mortality according to statin use

Group	Delirium					30-day mortality				
	No. of events/No. (%)	Crude.OR_95CI	*p*	adj.OR_95CI	*p*	No. of events/No. (%)	crude.OR_95CI	*p*	adj.OR_95CI	*p*
No statin use	237/1241 (19.1)	1 (Ref)		1 (Ref)		269/1241 (21.7)	1 (Ref)		1 (Ref)	
Statin use after ICU	68/644 (10.6)	0.5 (0.37–0.67)	< 0.001	0.55 (0.4–0.75)	**< 0.001**	107/644 (16.6)	0.72 (0.56–0.92)	0.009	0.85 (0.63–1.15)	0.287
Statin use before and after ICU	131/840 (15.5)	0.78 (0.62–0.99)	0.04	0.76 (0.58–1)	**0.047**	116/840 (13.8)	0.58 (0.46–0.73)	< 0.001	0.55 (0.41–0.73)	**< 0.001**

Adjusted model in delirium was adjusted for age, SBP, DBP, HR, SpO_2,_ CAD, asthma, liver disease, ACEI/ARB, antibiotic, glucocorticoids, HGB, PLT, sodium and calcium, norepinephrine, vasopressin, epinephrine, SAPII, OASIS, Los-ICU. Adjusted model in 30-day mortality was adjusted for age, SBP, DBP, HR, RR, SpO_2,_ CHF, asthma, Diabetes, liver disease, ACEI/ARB, β-blockers, antibiotic, glucocorticoids, WBC, HGB, potassium and sodium, norepinephrine, vasopressin, epinephrine, CCI, SAPII, OASIS, Los-ICU

### Subgroup analyses

**Fig. 3:**
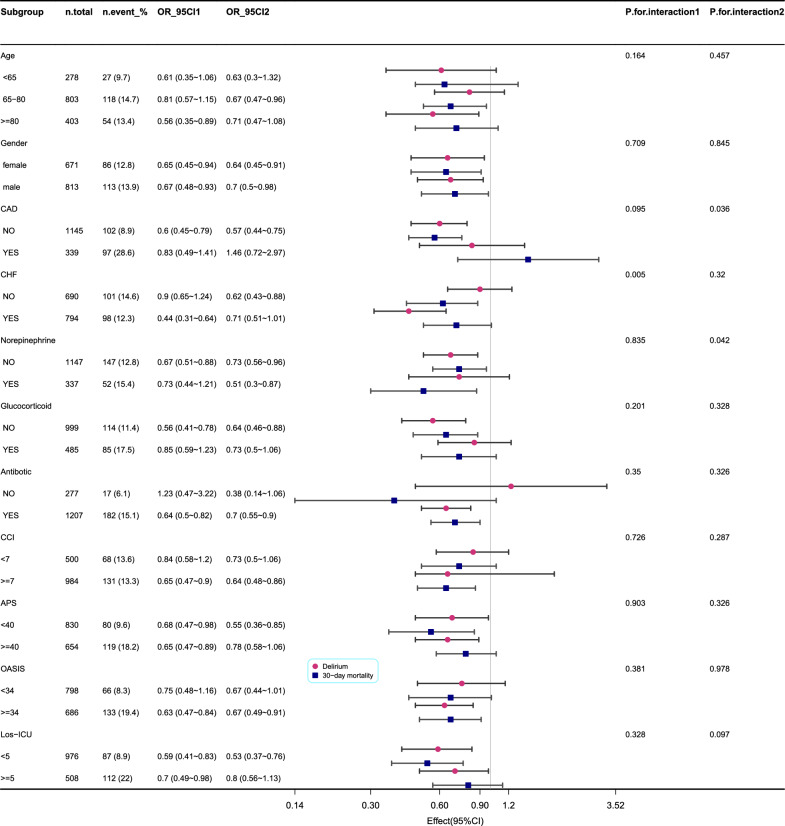
Subgroup analysis evaluating the relationship between statin exposure and delirium and 30-day mortality in patients with COPD

As shown in Fig. 3 (the original form is in Additional file 1: Tables S3 and S4), we found that patients with CHF showed an interaction between statin exposure and delirium (*p* = 0.005). Patients with CAD and expose to norepinephrine showed an interaction between statin exposure and 30-day mortality (*p* = 0.036 and *p* = 0.042). The p-value for the interaction in the other subgroups showed no interaction with delirium or mortality at 30 days.

## Discussion

In this observational study of patients with COPD, we used PSM and found that statin administration after admission to the ICU was significantly associated with a reduced risk of delirium regardless of whether statin was used or not before ICU admission. Statin used both before and after ICU was significantly associated with a reduced risk of 30-day mortality. To our knowledge, this is the first observational study on the association between statin exposure and delirium and 30-day mortality in patients with COPD. We subgrouped the timing of statin use with respect to whether the statin was used before or after ICU admission. The results demonstrated that the earlier the statin was used the more beneficial it was for 30-day mortality in patients with COPD.

Previous studies have found that statins reduce delirium in critically ill patients in the ICU [7, 20], but there have been no studies on statins in ICU patients with COPD. Although COPD has been found to increase the incidence of postoperative delirium in patients undergoing CABG [4], delirium is more common in patients with COPD combined with respiratory failure undergoing mechanical ventilation [3]. In our study, the use of statins in the ICU significantly reduced the incidence of delirium, and a subgroup analysis found that statins had a more significant effect in patients with CHF which is consistent with the study we done before [21]. Patients with CHF have higher rates of statin using (53.5% to 34%), especially they are used before ICU admission (58.7%). The role of statins in the management of people with cardiovascular disease is well understood.

In our study, 54.5% of patients with COPD received statins, and more than half of the patients were taking statins. Several studies have found that statins reduce hospitalization rates and acute exacerbations in patients with COPD [22–24] but do not influence 30-day or 1-year mortality [22] nor are they associated with the occurrence of COPD in the adult population [24]. However, it is interesting that observational studies have found that statins reduce COPD mortality [11], while a RCT did not find this effect of statins [12]. According to a previous retrospective analysis [25] of 574 individuals from the Copenhagen General Population Study, statin use was associated with a reduced probability of exacerbations only in individuals with COPD of the general population, but not in those with the most severe COPD without cardiovascular comorbidity. However, it is unknown whether the effects of statin on mortality are related to cardiovascular disease. Our subgroup analysis found that statins did not reduce mortality in patients with COPD with CAD.

COPD is considered a chronic systemic inflammatory syndrome that can often be accompanied by impaired blood oxygenation, and both inflammation and impaired blood oxygenation can lead to an increased incidence of delirium in patients with COPD. Decreased lung function is associated with increased oxidative stress and inflammation, and studies have shown that statins reduce the levels of inflammatory markers such as C-reactive protein (CRP) and interleukin-6 (IL-6) in COPD patients [26] and slow the decline in lung function [27]. Statins have been prescribed for the primary prevention of atherosclerotic cardiovascular disease because they effectively lower low-density lipoprotein (LDL) cholesterol levels, and a recent study found that the use of atorvastatin relieved cerebral vasospasm and mediated structural and functional remodeling of vascular endothelial cells [28], which may be related to the fact that statins can prevent delirium and mortality.

This study has some limitations. First, baseline data before admission to the MIMIC-IV database may be incomplete, which could have affected specific data relative to delirium regarding preoperative cognitive status, psychiatric history, and educational level. We excluded dementia and MIC, because these patients are prone to delirium [29, 30]. Second, we did not classify the type of statin used, although lipophilic statins are found to be more effective against acute exacerbations of COPD in patients with cardiovascular disease [24]. Third, our study was a retrospective study. Although we used PSM to control for confounding, residual confounders cannot be completely excluded. Finally, we could not assess the actual medicinal dosage or if patients admitted with an acute illness and the rounding physician decides to increase dosage after ICU admission. Large sample studies are warranted.

## Conclusions

Statin use is associated with a lower incidence of delirium and 30-day mortality in patients with COPD in the ICU. Continued statin use after hospital admission may be important in reducing mortality.

### Supplementary Information

**Additional file 1: Table S1.** Univariate regression analysis for delirium. **Table S2.** Univariate regression analysis for 30-day mortality. **Table S3.** Subgroup analysis of the relationship between statin exposure and delirium in patients with COPD. **Table S4.** Subgroup analysis of the relationship between statin exposure and 30-day mortality in patients with COPD. **Table S5.** Baseline characteristics according statin use before or after ICU.

## Data Availability

Publicly available datasets were analyzed in this study. These data can be found at https://physionet.org/content/mimiciv/2.2.

## Abbreviations

COPD: Chronic obstructive pulmonary disease
ICU: Intensive care unit
MIMIC-IV: Medical Information Mart for Intensive Care-IV
PSM: Propensity score matching
CI: Confidence interval
IPW: Inverse probability weighting
MCI: Mild cognitive impairment
