# Supplementary material for: Association between statin use on delirium and 30-day mortality in patients with chronic obstructive pulmonary disease in the intensive care unit
Source: Eur J Med Res. 2023 Dec 8;28:572. doi: 10.1186/s40001-023-01551-3 (PMC10704755; doi:10.1186/s40001-023-01551-3)
Supplement: Supplementary file 1 — Additional file 1: Table S1. Univariate regression analysis for delirium. Table S2. Univariate regression analysis for 30-day mortality. Table S3. Subgroup analysis of the relationship between statin exposure and delirium in patients with COPD. Table S4. Subgroup analysis of the relationship between statin exposure and 30-day mortality in patients with COPD. Table S5. Baseline characteristics according statin use before or after ICU. [file 40001_2023_1551_MOESM1_ESM.doc]

**Additional file**

**Association Between Statin Use on Delirium and 30-day Mortality in Patients With Chronic Obstructive Pulmonary Disease**

Jiangling Xia^1^ Leilei Wang^2^ Chunhuan Hu^3^ Yuzhu Zhang^1*^

1 Department of Anesthesiology,Zibo Central Hospital, Zibo,Shandong,China

2 School of Architecture and Engineering,Zibo Vocational Institute,Zibo,Shan Dong,China

3 Department of Anesthesiology,The Second Affiliated Hospital of Mudanjiang Medical College,Mudanjiang,Heilongjiang,China

**Additional file Tables**

**Table S1** univariate regression analysis for delirium

| Variable | OR_95CI | P_value |
| --- | --- | --- |
| Age(year) | 1 (0.99~1.01) | 0.82 |
| Gender(male) | 1.07 (0.87~1.31) | 0.527 |
| SBP(mmHg) | 1 (0.99~1) | 0.287 |
| DBP(mmHg) | 0.99 (0.98~1) | **0.008** |
| HR( beats/min) | 1.01 (1~1.01) | **0.034** |
| RR( beats/min) | 1.02 (0.99~1.05) | 0.201 |
| Spo2(%) | 1.03 (0.99~1.08) | 0.157 |
| Asthma | 0.14 (0.04~0.43) | **0.001** |
| CAD | 2.95 (2.34~3.73) | **<0.001** |
| CHF | 0.99 (0.81~1.22) | 0.953 |
| PVD | 1.06 (0.82~1.37) | 0.662 |
| CVD | 1.08 (0.82~1.42) | 0.595 |
| Diabetes | 1.02 (0.83~1.27) | 0.833 |
| Liver desease | 2.01 (1.53~2.66) | <**0.001** |
| renal disease | 0.92 (0.72~1.17) | 0.492 |
| malignant cancer | 0.8 (0.6~1.08) | **0.144** |
| WBC(10^9/L) | 1 (1~1.01) | 0.332 |
| HGB(g/L) | 1.06 (1.01~1.11) | **0.011** |
| PLT(10^9/L) | 1 (1~1) | **0.112** |
| Glucose | 1 (1~1) | **0.096** |
| Calcium(mg/dL) | 0.89 (0.77~1.03) | **0.113** |
| Sodium(mmol/L) | 1.05 (1.03~1.07) | **<0.001** |
| Potassium(mmol/L) | 0.94 (0.83~1.06) | 0.287 |
| Aniongap(mmol/L) | 1.01 (0.99~1.04) | 0.232 |
| Glucocorticoid | 1.4 (1.14~1.73) | **0.002** |
| Antibotic | 3.53 (2.34~5.33) | **<0.001** |
| Vasopressin | 1.3 (0.95~1.78) | **0.103** |
| Norepinephrine | 1.19 (0.93~1.52) | **0.159** |
| Epinephrine | 1.47 (0.96~2.26) | **0.079** |
| ACEI/ARB | 0.65 (0.47~0.89) | **0.007** |
| β-blocker | 1.03 (0.84~1.27) | 0.747 |
| VENT | 1.03 (0.83~1.28) | 0.795 |
| CCI | 0.99 (0.95~1.03) | 0.584 |
| SAPII | 1.03 (1.02~1.04) | **<0.001** |
| OASIS | 1.07 (1.06~1.08) | **<0.001** |
| Los-ICU | 1.06 (1.04~1.07) | **<0.001** |

Bolded items are p<0.2.

**Abbreviations**:SBP:Systolic blood pressure.DBP:Diastolic blood pressure.HR:heart rate .RR: respiratory rate, CAD:coronary artery disease.CHF:congestive heart failure.PVD:peripheral vascular disease.CVD:cerebrovascular disease.HGB:Hemoglobin.WB:white blood Cell count.PLT:platelets.ACEI/ARB:Angiotensin-converting enzyme inhibitors/Angiotensin II inhibitors.VENT:Mechanical ventilation CCI:Charlson Comorbidity Index.SAPS II: Simplified Acute Physiology Score.OASIS:Oxford acute severity of illness score. Los:length of stay.

**Table S2.**univariate regression analysis for 30-day mortality

| Variable | OR_95CI | P_value |
| --- | --- | --- |
| Age(year) | 1.04 (1.03~1.05) | **<0.001** |
| Gender(male) | 0.93 (0.77~1.14) | 0.492 |
| SBP(mmHg) | 0.99 (0.98~0.99) | **<0.001** |
| DBP(mmHg) | 0.99 (0.98~1) | **0.009** |
| HR( beats/min) | 1.02 (1.02~1.03) | **<0.001** |
| RR( beats/min) | 1.11 (1.08~1.14) | **<0.001** |
| Spo2(%) | 0.94 (0.9~0.98) | **0.002** |
| Asthma | 0.57 (0.32~1.03) | **0.062** |
| CAD | 1.01 (0.78~1.31) | 0.929 |
| CHF | 1.44 (1.18~1.75) | **<0.001** |
| PVD | 1.15 (0.9~1.46) | 0.272 |
| CVD | 1.07 (0.82~1.4) | 0.603 |
| Diabetes | 0.79 (0.64~0.97) | **0.025** |
| Liver desease | 1.86 (1.42~2.44) | **<0.001** |
| Renal disease | 1.58 (1.28~1.95) | **<0.001** |
| Malignant cancer | 2.21 (1.75~2.79) | **<0.001** |
| WBC(10^9/L) | 1.02 (1.01~1.03) | **<0.001** |
| HGB(g/L) | 0.87 (0.83~0.91) | **<0.001** |
| PLT(10^9/L) | 1 (1~1) | 0.202 |
| Glucose(mg/dL) | 1 (1~1) | **0.004** |
| Calcium(mg/dL) | 1.01 (0.88~1.15) | 0.933 |
| Sodium(mmol/L) | 0.98 (0.96~1) | **0.059** |
| Potassium(mmol/L) | 1.18 (1.06~1.31) | **0.002** |
| Aniongap(mmol/L) | 1.08 (1.06~1.11) | **<0.001** |
| Glucocorticoid | 1.93 (1.58~2.35) | **<0.001** |
| Antibotic | 3.04 (2.11~4.37) | **<0.001** |
| Vasopressin | 1.34 (0.99~1.8) | **0.057** |
| Norepinephrine | 1.44 (1.15~1.81) | **0.001** |
| Epinephrine | 1.8 (1.21~2.67) | **0.004** |
| ACEI/ARB | 0.3 (0.2~0.44) | **<0.001** |
| Β-blocker | 0.66 (0.54~0.8) | **<0.001** |
| VENT | 1.11 (0.9~1.37) | 0.317 |
| CCI | 1.25 (1.2~1.3) | **<0.001** |
| SAPII | 1.06 (1.05~1.07) | **<0.001** |
| OASIS | 1.08 (1.07~1.09) | **<0.001** |
| Los-ICU | 1.02 (1~1.03) | **0.01** |

Bolded items arep p<0.2.

**Abbreviations**:SBP:Systolic blood pressure.DBP:Diastolic blood pressure.HR:heart rate .RR: respiratory rate, CAD:coronary artery disease.CHF:congestive heart failure.PVD:peripheral vascular disease.CVD:cerebrovascular disease.HGB:Hemoglobin.WB:white blood Cell count.PLT:platelets.ACEI/ARB:Angiotensin-converting enzyme inhibitors/Angiotensin II inhibitors.VENT:Mechanical ventilation CCI:Charlson Comorbidity Index.SAPS II: Simplified Acute Physiology Score.OASIS:Oxford acute severity of illness score.Los:length of stay.

**Table S3**.Subgroup analysis of the relationship between statin exposure and delirium in patients with COPD.

|  |  |  |  |  |  |  |  |
| --- | --- | --- | --- | --- | --- | --- | --- |
| Subgroup | n.total | n.event_% | OR_95CI | P.for.interaction | OR | OR.95CI.Low | OR.95CI.Up |
| Age |  |  |  | 0.164 |  |  |  |
| <65 | 278 | 27 (9.7) | 0.61 (0.35~1.06) |  | 0.61 | 0.35 | 1.06 |
| 65-80 | 803 | 118 (14.7) | 0.81 (0.57~1.15) |  | 0.81 | 0.57 | 1.15 |
| >80 | 403 | 54 (13.4) | 0.56 (0.35~0.89) |  | 0.56 | 0.35 | 0.89 |
| Gender |  |  |  | 0.709 |  |  |  |
| female | 671 | 86 (12.8) | 0.65 (0.45~0.94) |  | 0.65 | 0.45 | 0.94 |
| male | 813 | 113 (13.9) | 0.67 (0.48~0.93) |  | 0.67 | 0.48 | 0.93 |
| CAD |  |  |  | 0.095 |  |  |  |
| NO | 1145 | 102 (8.9) | 0.6 (0.45~0.79) |  | 0.6 | 0.45 | 0.79 |
| YES | 339 | 97 (28.6) | 0.83 (0.49~1.41) |  | 0.83 | 0.49 | 1.41 |
| CHF |  |  |  | **0.005** |  |  |  |
| NO | 690 | 101 (14.6) | 0.9 (0.65~1.24) |  | 0.9 | 0.65 | 1.24 |
| YES | 794 | 98 (12.3) | 0.44 (0.31~0.64) |  | 0.44 | 0.31 | 0.64 |
| Norepinephrine |  |  |  | 0.835 |  |  |  |
| NO | 1147 | 147 (12.8) | 0.67 (0.51~0.88) |  | 0.67 | 0.51 | 0.88 |
| YES | 337 | 52 (15.4) | 0.73 (0.44~1.21) |  | 0.73 | 0.44 | 1.21 |
| Glucocorticoid |  |  |  | 0.201 |  |  |  |
| NO | 999 | 114 (11.4) | 0.56 (0.41~0.78) |  | 0.56 | 0.41 | 0.78 |
| YES | 485 | 85 (17.5) | 0.85 (0.59~1.23) |  | 0.85 | 0.59 | 1.23 |
| Antibotic |  |  |  | 0.35 |  |  |  |
| NO | 277 | 17 (6.1) | 1.23 (0.47~3.22) |  | 1.23 | 0.47 | 3.22 |
| YES | 1207 | 182 (15.1) | 0.64 (0.5~0.82) |  | 0.64 | 0.5 | 0.82 |
| CCI |  |  |  | 0.726 |  |  |  |
| <7 | 500 | 68 (13.6) | 0.84 (0.58~1.2) |  | 0.84 | 0.58 | 1.2 |
| >=7 | 984 | 131 (13.3) | 0.65 (0.47~0.9) |  | 0.65 | 0.47 | 1.9 |
| SAPII |  |  |  | 0.903 |  |  |  |
| <40 | 830 | 80 (9.6) | 0.68 (0.47~0.98) |  | 0.68 | 0.47 | 0.98 |
| >=40 | 654 | 119 (18.2) | 0.65 (0.47~0.89) |  | 0.65 | 0.47 | 0.89 |
| OASIS |  |  |  | 0.381 |  |  |  |
| <34 | 798 | 66 (8.3) | 0.75 (0.48~1.16) |  | 0.75 | 0.48 | 1.16 |
| >=34 | 686 | 133 (19.4) | 0.63 (0.47~0.84) |  | 0.63 | 0.47 | 0.84 |
| Los-ICU |  |  |  | 0.328 |  |  |  |
| <5 | 976 | 87 (8.9) | 0.59 (0.41~0.83) |  | 0.59 | 0.41 | 0.83 |
| >=5 | 508 | 112 (22) | 0.7 (0.49~0.98) |  | 0.7 | 0.49 | 0.98 |

Bolded items arep p<0.05

**Abbreviations**:CAD:coronary artery disease.CHF:congestive heart failure.CCI:Charlson Comorbidity Index.

**Table S4.**Subgroup analysis of the relationship between statin exposure and 30-day mortality in patients with COPD.

| Subgroup | n.total | n.event_% | OR_95CI | P.for.interaction | OR | OR.95CI.Low | OR.95CI.Up |
| --- | --- | --- | --- | --- | --- | --- | --- |
| Age |  |  |  | 0.457 |  |  |  |
| <65 | 278 | 17 (6.1) | 0.63 (0.3~1.32) |  | 0.63 | 0.47 | 1.32 |
| 65-80 | 803 | 107 (13.3) | 0.67 (0.47~0.96) |  | 0.67 | 0.51 | 0.96 |
| >80 | 403 | 99 (24.6) | 0.71 (0.47~1.08) |  | 0.71 | 0.47 | 1.08 |
| Gender |  |  |  | 0.845 |  |  |  |
| female | 671 | 102 (15.2) | 0.64 (0.45~0.91) |  | 0.64 | 0.45 | 0.91 |
| male | 813 | 121 (14.9) | 0.7 (0.5~0.98) |  | 0.7 | 0.5 | 0.98 |
| CAD |  |  |  | 0.036 |  |  |  |
| NO | 1145 | 165 (14.4) | 0.57 (0.44~0.75) |  | 0.57 | 0.44 | 0.75 |
| YES | 339 | 58 (17.1) | 1.46 (0.72~2.97) |  | 1.46 | 0.72 | 2.97 |
| CHF |  |  |  | 0.32 |  |  |  |
| NO | 690 | 78 (11.3) | 0.62 (0.43~0.88) |  | 0.62 | 0.43 | 0.88 |
| YES | 794 | 145 (18.3) | 0.71 (0.51~1.01) |  | 0.71 | 0.51 | 1.01 |
| Norepinephrine |  |  |  | 0.042 |  |  |  |
| NO | 1147 | 167 (14.6) | 0.73 (0.56~0.96) |  | 0.73 | 0.56 | 0.96 |
| YES | 337 | 56 (16.6) | 0.51 (0.3~0.87) |  | 0.51 | 0.3 | 0.87 |
| Glucocorticoid |  |  |  | 0.328 |  |  |  |
| NO | 999 | 110 (11) | 0.64 (0.46~0.88) |  | 0.64 | 0.46 | 0.88 |
| YES | 485 | 113 (23.3) | 0.73 (0.5~1.06) |  | 0.73 | 0.5 | 1.06 |
| Antibotic |  |  |  | 0.326 |  |  |  |
| NO | 277 | 15 (5.4) | 0.38 (0.14~1.06) |  | 0.38 | 0.14 | 1.06 |
| YES | 1207 | 208 (17.2) | 0.7 (0.55~0.9) |  | 0.7 | 0.55 | 0.9 |
| CCI |  |  |  | 0.287 |  |  |  |
| <7 | 500 | 43 (8.6) | 0.73 (0.5~1.06) |  | 0.73 | 0.5 | 1.06 |
| >=7 | 984 | 180 (18.3) | 0.64 (0.48~0.86) |  | 0.64 | 0.48 | 0.86 |
| SAPII |  |  |  | 0.326 |  |  |  |
| <40 | 830 | 59 (7.1) | 0.55 (0.36~0.85) |  | 0.55 | 0.36 | 0.85 |
| >=40 | 654 | 164 (25.1) | 0.78 (0.58~1.06) |  | 0.78 | 0.58 | 1.06 |
| OASIS |  |  |  | 0.978 |  |  |  |
| <34 | 798 | 74 (9.3) | 0.67 (0.44~1.01) |  | 0.67 | 0.44 | 1.01 |
| >=34 | 686 | 149 (21.7) | 0.67 (0.49~0.91) |  | 0.67 | 0.49 | 0.91 |
| Los-ICU |  |  |  | 0.097 |  |  |  |
| <5 | 976 | 109 (11.2) | 0.53 (0.37~0.76) |  | 0.53 | 0.37 | 0.76 |
| >=5 | 508 | 114 (22.4) | 0.8 (0.56~1.13) |  | 0.8 | 0.56 | 1.13 |

Bolded items arep p<0.05

**Abbreviations**:CAD:coronary artery disease.CHF:congestive heart failure.CCI:Charlson Comorbidity Index.

**Table S5.** Baseline characteristics according statin use before or after ICU.

| Variables | Total (n = 2725) | non-statins | statins after ICU | statins before and after ICU | p |
| --- | --- | --- | --- | --- | --- |
|  |  | (n = 1241) | (n = 644) | (n = 840) |  |
| **Number** |  |  |  |  |  |
| Age(year) | 72.0 (64.0, 80.0) | 69.0 (61.0, 78.0) | 73.0 (66.0, 80.0) | 73.5 (67.0, 81.0) | < 0.001 |
| Gender(male) | 1456 (53.4) | 643 (51.8) | 357 (55.4) | 456 (54.3) | 0.274 |
| Los-ICU | 3.0 (2.0, 6.0) | 4.0 (2.0, 7.0) | 3.0 (2.0, 5.0) | 4.0 (2.0, 6.0) | < 0.001 |
| Hospital days | 9.0 (6.0, 15.0) | 10.0 (6.0, 17.0) | 7.0 (5.0, 10.0) | 12.0 (8.0, 18.0) | < 0.001 |
| **Vitals** |  |  |  |  |  |
| SBP(mmHg) | 117.0 ± 16.0 | 116.6 ± 15.6 | 118.2 ± 17.1 | 116.8 ± 15.8 | 0.113 |
| DBP(mmHg) | 61.9 ± 10.8 | 63.1 ± 11.0 | 62.1 ± 10.6 | 60.0 ± 10.5 | < 0.001 |
| HR( beats/min) | 85.9 ± 15.6 | 87.7 ± 16.4 | 83.3 ± 14.5 | 85.2 ± 14.9 | < 0.001 |
| RR( beats/min) | 19.8 ± 3.7 | 19.9 ± 3.9 | 19.7 ± 3.5 | 19.8 ± 3.5 | 0.453 |
| Spo2(%) | 96.2 ± 2.3 | 96.2 ± 2.3 | 96.1 ± 2.2 | 96.2 ± 2.2 | 0.773 |
| **Comorbidities**, n (%) |  |  |  |  |  |
| Asthma | 114 ( 4.2) | 55 (4.4) | 27 (4.2) | 32 (3.8) | 0.785 |
| CAD | 456 (16.7) | 117 (9.4) | 115 (17.9) | 224 (26.7) | < 0.001 |
| CHF | 1216 (44.6) | 422 (34) | 301 (46.7) | 493 (58.7) | < 0.001 |
| PVD | 517 (19.0) | 168 (13.5) | 141 (21.9) | 208 (24.8) | < 0.001 |
| CVD | 433 (15.9) | 161 (13) | 124 (19.3) | 148 (17.6) | < 0.001 |
| Diabetes | 938 (34.4) | 315 (25.4) | 252 (39.1) | 371 (44.2) | < 0.001 |
| Liver desease | 314 (11.5) | 219 (17.6) | 40 (6.2) | 55 (6.5) | < 0.001 |
| Renal disease | 692 (25.4) | 215 (17.3) | 185 (28.7) | 292 (34.8) | < 0.001 |
| Malignant cancer | 446 (16.4) | 255 (20.5) | 80 (12.4) | 111 (13.2) | < 0.001 |
| **Laboratory events** |  |  |  |  |  |
| WBC(10^9/L) | 14.7 ± 11.2 | 15.0 ± 14.1 | 14.7 ± 7.2 | 14.4 ± 8.5 | 0.461 |
| HGB(g/L) | 11.3 ± 2.2 | 11.5 ± 2.3 | 11.5 ± 2.1 | 11.0 ± 2.0 | < 0.001 |
| PLT(10^9/L) | 209.0 (156.0, 277.0) | 206.0 (148.0, 284.0) | 210.0 (159.0, 266.0) | 211.0 (161.0, 278.5) | 0.371 |
| Glucose(mg/dL) | 147.0 (119.0, 196.0) | 144.0 (118.0, 190.0) | 151.5 (123.0, 204.0) | 146.0 (118.8, 197.0) | 0.024 |
| Calcium(mg/dL) | 8.6 ± 0.7 | 8.6 ± 0.8 | 8.6 ± 0.7 | 8.6 ± 0.7 | 0.137 |
| Sodium(mmol/L) | 139.6 ± 4.9 | 139.5 ± 5.3 | 140.0 ± 4.7 | 139.5 ± 4.6 | 0.184 |
| Potassium(mmol/L) | 4.7 ± 0.9 | 4.7 ± 0.9 | 4.8 ± 0.9 | 4.7 ± 0.8 | 0.066 |
| Aniongap(mmol/L) | 16.4 ± 4.5 | 16.5 ± 4.7 | 16.5 ± 4.7 | 16.1 ± 4.1 | 0.048 |
| **Treatment**, n (%) |  |  |  |  |  |
| Glucocorticoid | 968 (35.5) | 483 (38.9) | 193 (30) | 292 (34.8) | < 0.001 |
| Antibotic | 2280 (83.7) | 1073 (86.5) | 471 (73.1) | 736 (87.6) | < 0.001 |
| Vasopressin | 284 (10.4) | 114 (9.2) | 64 (9.9) | 106 (12.6) | 0.038 |
| Norepinephrine | 575 (21.1) | 238 (19.2) | 132 (20.5) | 205 (24.4) | 0.015 |
| Epinephrine | 130 ( 4.8) | 56 (4.5) | 29 (4.5) | 45 (5.4) | 0.631 |
| ACEI/ARB | 416 (15.3) | 122 (9.8) | 131 (20.3) | 163 (19.4) | < 0.001 |
| β-blocker | 1437 (52.7) | 545 (43.9) | 378 (58.7) | 514 (61.2) | < 0.001 |
| VENT | 873 (32.0) | 387 (31.2) | 203 (31.5) | 283 (33.7) | 0.461 |
| **Scores** |  |  |  |  |  |
| CCI | 7.4 ± 2.6 | 7.0 ± 2.6 | 7.6 ± 2.5 | 8.0 ± 2.4 | < 0.001 |
| SAPII | 39.8 ± 12.8 | 40.4 ± 14.0 | 38.3 ± 11.3 | 40.0 ± 11.7 | 0.003 |
| OASIS | 34.3 ± 9.0 | 35.5 ± 9.4 | 33.4 ± 8.5 | 33.2 ± 8.5 | < 0.001 |
| **Outcomes,**n (%) |  |  |  |  |  |
| Delirium | 436 (16.0) | 237 (19.1) | 68 (10.6) | 131 (15.6) | < 0.001 |
| 30-day mortality | 492 (18.1) | 269 (21.7) | 107 (16.6) | 116 (13.8) | < 0.001 |

**Notes**:Data are presented as mean (sd),medians [interquartile ranges] or numbers (percentages).

**Abbreviations**:SBP:Systolic blood pressure.DBP:Diastolic blood pressure.HR:heart rate .RR: respiratory rate, CAD:coronary artery disease.CHF:congestive heart failure.PVD:peripheral vascular disease.CVD:cerebrovascular disease.HGB:Hemoglobin.WB:white blood Cell count.PLT:platelets.ACEI/ARB:Angiotensin-converting enzyme inhibitors/Angiotensin II inhibitors.VENT:Mechanical ventilation CCI:Charlson Comorbidity Index.SAPS II: Simplified Acute Physiology Score.OASIS:Oxford acute severity of illness score.Los:length of stay.
